# Herpes Simplex Virus: The Hostile Guest That Takes Over Your Home

**DOI:** 10.3389/fmicb.2020.00733

**Published:** 2020-05-07

**Authors:** Anwesha Banerjee, Smita Kulkarni, Anupam Mukherjee

**Affiliations:** Division of Virology, Indian Council of Medical Research-National AIDS Research Institute, Pune, India

**Keywords:** herpes, organelles, autophagy, apoptosis, encephalitis, therapeutics

## Abstract

Alpha (α)-herpesviruses (HSV-1 and HSV-2), like other viruses, are obligate intracellular parasites. They hijack the cellular machinery to survive and replicate through evading the defensive responses by the host. The viral genome of herpes simplex viruses (HSVs) contains viral genes, the products of which are destined to exploit the host apparatus for their own existence. Cellular modulations begin from the entry point itself. The two main gateways that the virus has to penetrate are the cell membrane and the nuclear membrane. Changes in the cell membrane are triggered when the glycoproteins of HSV interact with the surface receptors of the host cell, and from here, the components of the cytoskeleton take over. The rearrangement in the cytoskeleton components help the virus to enter as well as transport to the nucleus and back to the cell membrane to spread out to the other cells. The entire carriage process is also mediated by the motor proteins of the kinesin and dynein superfamily and is directed by the viral tegument proteins. Also, the virus captures the cell’s most efficient cargo carrying system, the endoplasmic reticulum (ER)–Golgi vesicular transport machinery for egress to the cell membrane. For these reasons, the host cell has its own checkpoints where the normal functions are halted once a danger is sensed. However, a cell may be prepared for the adversities from an invading virus, and it is simply commendable that the virus has the antidote to these cellular strategies as well. The HSV viral proteins are capable of limiting the use of the transcriptional and translational tools for the cell itself, so that its own transcription and translation pathways remain unhindered. HSV prefers to constrain any self-destruction process of the cell—be it autophagy in the lysosome or apoptosis by the mitochondria, so that it can continue to parasitize the cell for its own survival. This review gives a detailed account of the significance of compartmentalization during HSV pathogenesis. It also highlights the undiscovered areas in the HSV cell biology research which demand attention for devising improved therapeutics against the infection.

## Introduction

Alpha (α)-herpesviruses are DNA viruses belonging to the family *Herpesviridae; herpein* meaning “to creep.” Their members belong to one of the genera: *Iltovirus, Mardivirus, Scutavirus, Simplexvirus, and Varicellovirus*. The virions of α-herpesviruses are encased within a lipid bilayer envelope and capable of productive lysis as well as establishing a latent infection that is reactivable. The human α-herpesviruses, consisting of herpes simplex virus (HSV-1 and HSV-2) and varicella zoster virus (VZV), have a wide range of vertebrate and invertebrate hosts to infect (Pellett and Roizman, 2013). Infection from HSV-1 causes corneal keratitis and/or cold sores at the orolabial region, whereas HSV-2 infection is mainly accountable for lesions at the genitalia (Shukla and Spear, 2001). In some exceptional cases, HSV-1 can cause genital herpes and HSV-2 could be responsible for oral herpes as well. HSV, although asymptomatic in many cases, causes viral shedding when the viral load is high and direct contact with infected patients through mucus and other body fluids results in the acquisition of infection (Fatahzadeh and Schwartz, 2007). Therefore, HSV-2 could be transmitted from mother to newborn during child birth *via* an infected birth canal (Anzivino et al., 2009). Most severe manifestations of HSV are encephalitis, meningitis, and blindness (Connolly et al., 2011). In developed countries, HSV-1 is marked as the major cause of corneal blindness and encephalitis through viruses (Herpetic Eye Disease Study Group, 1998; Shoji et al., 2002). Infection *via* HSV can cause direct destruction of the cell *via* lysis or can hide itself from the attacks of the host immune system by establishing latency (Whitley and Roizman, 2001) in a cell type-specific manner. HSV-1 and HSV-2 cause latency in the sensory neurons and the ganglia. By the establishment of latency, HSV can avoid encountering the antiviral drugs such as acyclovir and its analogs (James and Prichard, 2014).

Herpes simplex viruses are enveloped double-stranded DNA viruses. The outer envelope consists of 16 membrane proteins, out of which 12 are glycoproteins (Campadelli-Fiume et al., 2000; Mettenleiter, 2004; Diefenbach et al., 2008). These glycoproteins (gB, gC, gD, gE, gG, gH, gI, gJ, gK, gL, gM, and gN) mainly assist the entry of the virus into host cells. Below the envelope is the tegument which contains about 22 viral proteins (VPs). Beneath the tegument lies the icosahedral capsid encapsulating the HSV genome. The capsid has 162 capsomeres and six VPs on its surface (Diefenbach et al., 2008). The innermost core of the virus particle is the HSV genome of about 152 kB, from where at least 74 genes are encoded (McGeoch et al., 2006). From the beginning of the encounter of the virus with the host cell, HSV is ready with a strategized plan to divert the components of the host cell toward its pathogenesis to establish a productive infection. At present, our knowledge of understanding toward organelle dynamics during HSV infections is still at its infancy. In this review, we briefly summarize those mechanistic processes of HSV toward the various cellular organelles that lead to an extensive host cellular reorganization for prosperous establishment of the viral life cycle. This review will serve as a connection between the two most important sections, HSV virology and host cellular biology, which lead toward the development of new research avenues. The review goes about the events that take place at the cell organelles during an HSV infection.

## The Cell Membrane

### The “Main Gateway” to Herpes Simplex Virus Entry

**FIGURE 1:**
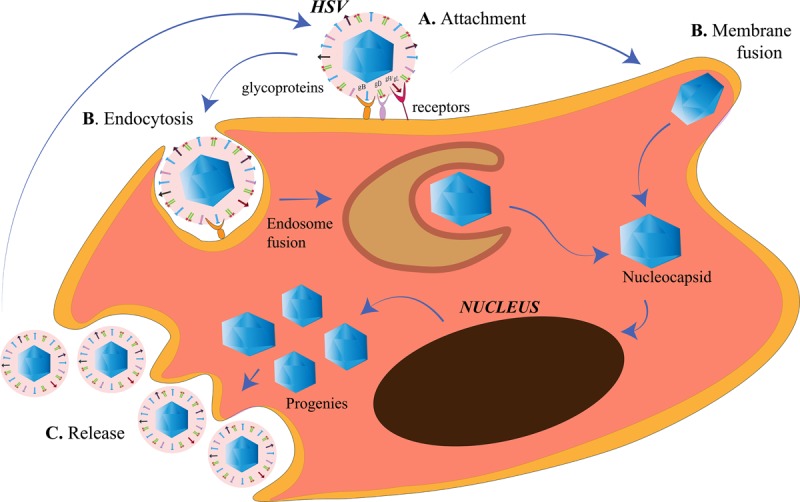
Herpes simplex virus (HSV) entry and release at the cell membrane. **(A)** *Attachment*. The HSV envelope glycoproteins gB, gD, and gH/gL interact with the receptors on the cell membrane. **(B)** *Penetration*. Entry of HSV into the cell takes place either by membrane fusion, where the membrane of the virus and the cell fuse for the cellular uptake of the nucleocapsid only or, by endocytosis, where the enveloped virus is engulfed in an endosome followed by endosome fusion to release the nucleocapsids. The nucleocapsids enter into the nucleus for viral replication. **(C)** *Release*. The HSV progenies are released into the cytoplasm, acquire the membrane from the host cell in a process called *budding*.

The membrane of a cell acts as the fence of the cell, giving it the characteristic shape. It also acts as the “doorway” for entry as well as exit of substances from the cell. The cell membrane of the target cells of HSV, like any other animal cell, is semipermeable that is selective to the contents moving in and out of the cell. HSV is capable of targeting such cells because it has adapted itself to do so in the course of evolution (Karasneh and Shukla, 2011). HSV is an enveloped virus, and its envelop is derived from the cell membrane of the host cell it infects during the process of “budding out.” Although membrane fusion for entry is a speciality of the enveloped viruses due to the presence of a lipid bilayer around them, HSV is capable of exploiting other routes of entry as well (Wittels and Spear, 1991; Clement et al., 2006) (Figure 1). It is capable of introducing membrane disruptions by forming pores or fragmentations in the membrane to induce endocytosis (Wittels and Spear, 1991). The route of entry is cell-specific. The virus enters the epithelial cells *via* the endocytic pathway and the neuronal cells *via* the membrane fusion pathway (Nicola et al., 2005; Miranda-Saksena et al., 2018) (Figure 1). The factors that direct the virus to choose the entry route in a cell are unclear. However, this choice is highly dependent on the replication cycle of the virus. HSV uses the epithelial cells to establish its lytic phase and the neuronal cells to establish a lysogenic phase. However, HSV replication has been observed in neuronal cells as well. Increased viral replication (such as during reactivation) in neurons may lead to critical manifestations such as encephalitis (Kennedy and Steiner, 2013). There are three events occurring at the cell membrane of the target host cell, namely, attachment, penetration and release (Figure 1). All of these processes involve the crucial participation of viral glycoproteins.

### Attachment

An attachment process involving a receptor–ligand interaction is a common phenomenon for an HSV particle entering the cell, irrespective of the route of entry. The glycoproteins present on the surface of the HSV act as the ligands to the receptors present on the host cell surface to initiate the attachment process. There are 15 membrane proteins on the lipid envelope of HSV-1, of which 12 are glycosylated (Karasneh and Shukla, 2011). Of all glycosylated proteins or the glycoproteins (g), only four (gD, gH, gL, and gB) are essential for the entry process (Turner et al., 1998; Heldwein and Krummenacher, 2008). Glycoprotein B (gB), along with gC (a non-essential glycoprotein), is required for the attachment of the HSV to the heparan sulfate (HS) proteoglycans (HSPGs) on the cell surface (Heldwein and Krummenacher, 2008). After the attachment of gB to its receptor, gD binds to any one of its receptors, nectin-1 or herpes virus entry mediator (HVEM) or HS modified by the 3-OST family (3-OS-HS), to bring about a conformational change in itself (Agelidis and Shukla, 2015). Nectin-1 and nectin-2 are members of the immunoglobulin superfamily. Whereas HSV-2 entry can be mediated with both, nectin-2-mediated wild-type HSV-1 entry has not been observed yet (Krummenacher et al., 2004). HVEM is one of the members of tumor necrosis factor (TNF)-receptor superfamily that is mainly involved in facilitating the entry of HSV in T cells and some of the ocular epithelial cells. Although both nectin-1 and HVEM are receptors of gD, they have their own distinct pathways of HSV entry and block the entry of the virus through the other (Zhang et al., 2011). 3-OS-HS is the major receptor for HSV in the corneal fibroblasts where nectin-1 and HVEM have diminished expressions (Tiwari et al., 2006). The change in the conformation of gD is the displacement of the C-terminus after which the fusion-activating domain of gD is exposed (Gallagher et al., 2013). The changed gD conformation allows it to bind to a heterodimer of gH/gL (Fan et al., 2014). The gD/gH/gL complex then activates the fusogenic domain of gB to initiate the penetration process. The gD/gH/gL complex along with the gB is referred to as the core receptor-binding apparatus of the HSV (Atanasiu et al., 2013). It is also laudable the extent up to which the virus controls cell receptors, such that these proteins prioritize their role in the viral pathogenesis rather than their normal duty toward the host cell. gD of HSV is known to bind to the homodimerization interface of the nectin-1 so that nectin-1 is unable to function as a cell adhesion molecule (Zhang et al., 2011). Heparan sulfate (HS) and gB are also required for HSV surfing. HSV surfing is the phenomenon by which HSV-1 virions are known to transport to the cell bodies. This phenomenon is aided by the actin filaments of the cytoskeleton, whereas small Rho GTPase, Cdc42, is one of the regulators for the same (Oh et al., 2010). Once HSV has exploited the receptors for its entry, it is ready to penetrate the cell to establish an infection.

### Penetration

After the attachment of HSV on the host cell, the membrane of the virus and the host cell need to be fused. Fusogens are specialized glycoproteins on the viral surface that mediate the fusion of the two membranes by introducing conformational changes in the membranes. The fusogen is generally spring-loaded and is triggered when the virus lands on the correct target cell or the intracellular compartment such as the endosome. The activation of the fusogen is either receptor-dependent or dependent on the pH of the compartment (Mas and Melero, 2013). HSV-1 gB is a fusogen that mediates the membrane fusion to facilitate the penetration of the HSV into the host cell (Atanasiu et al., 2013).

HSV-1 and HSV-2 has been known to use the endocytic pathway to enter the host cells. Clement et al. (2006) demonstrated a new phagocytosis-like mechanism for HSV endocytosis. While approaching the epithelial cells, HSV-1 interacted with the membrane protrusions and was then engulfed by epithelium. The phagocytosis mechanism involved rearrangement of the cytoskeleton by the activation of Rho GTPases, followed by en-routing the virus in phagosome-like vesicles. The endocytosis was not clathrin-mediated because Eps15-deleted mutants were not affected for HSV-1 entry. Nectin or HVEM clustering in the phagosomes were also observed in HSV-1-infected cells, suggesting the contact between the envelope of the virus and that of the phagosomal membrane. Phagocytosis-mediated HSV uptake had since been a novel mechanism for HSV entry in epithelial cells (non-professional phagocytes). Efficient replication of HSV prepares the progenies and the cell for the release into the extracellular environment to spread and infect the neighborhood cells.

### Release

As in the case of the viral entry, the release of HSV-1 from the infected cells requires certain glycoproteins. The virus is willing to leave the hijacked cell in order to spread to the other uninfected cells to further increase its number of progeny viruses. For example, the gp heterodimer, gE/gI, redistributes itself to the cell junctions to facilitate viral spread to the other cells (Farnsworth and Johnson, 2006). In neuronal cells, the gE/gI helps the transport of the capsids from the cell body through the axons by bringing them close to the kinesin motor proteins for anterograde transport (Howard et al., 2012). Thus, gE/gI may also use the same mechanism for reactivation from latency by facilitating the spread of HSV from the cell body of the neurons to the epithelial cells (Howard et al., 2014). Also, gK was discovered to be responsible for the spread of HSV from the corneal epithelium to the neurons, suggesting that gK is important for the establishment of latency as corneal infection with HSV-1 that had a mutation in the N-terminus of gK, failed to infect the trigeminal ganglia in mice (David et al., 2008, 2012; Saied et al., 2014). Heparanase is an endoglycosidase that can degrade HS by catalyzing the cleavage of the β-(1,4)-glycosidic bond between the glucuronic acid and glucosamine residues of HS (Agelidis et al., 2017). HSV requires heparanase-1 (HPSE) to be released from the infected cells (Hadigal et al., 2015). HPSE loosens the binding between the virus and the receptor HS to be free from the cell. It is simply wondrous that HSV controls the cell in such a manner that the HPSE levels increase gradually after infection. This eventual increment in the HPSE levels is a preplanned strategy to shift the cell from the viral attachment to the HSV viral detachment state, so that the release of HSV is smooth. The released HSV progenies are capable of reinitiating the entry process in the nearby cells. The cytoskeleton takes over after the virus has entered the cells.

## Cytoskeleton

### The “Highways” for Herpes Simplex Virus Trafficking Within the Cell

The cytoskeleton is the backbone of the cell. It is an intracellular network of microfilaments, intermediate filaments, and microtubules and is capable of interacting with HSV (Feierbach et al., 2006). During HSV infection, the virus utilizes this network to enter and travel across the cell to the nucleus. In the nucleus, the viral replication is assisted by the microfilaments and assemble with a capsid before egress. The changes in the cytoskeleton structure either help in pathogenesis of HSV or counteract them. These cytoskeletal transformations in the cells can render them cancerous (Zheng et al., 2007).

### Herpes Simplex Virus Exploits the Microfilaments and the Microtubules for Retrograde/Anterograde Transport

Prior to HSV penetration in the cell, the virus comes across the actin filaments lying toward the cytosolic side of the cell membrane and those which are bound to the surface receptors. When HSV-1 gD interacts with the nectin-1 on the cell surface, the Rho GTPase signaling is activated, which further causes the rearrangement of the actin connected to them (Hoppe et al., 2006). Even during the HSV egress process, the interaction of non-muscle myosin IIA (NMIIA) with VP22, which is an HSV tegument protein, is vital for the virions that leave the cell to enter into the extracellular milieu (Conti and Adelstein, 2008; Wang et al., 2017). After successful entry into the cells, the microfilaments, along with the motor protein dynein, assist the HSV from the membrane to the nucleus. As reviewed by Wu et al. (2019), when HSV needs to spread itself to the other host cells, it utilizes the US3 protein kinase to phosphorylate RhoA to rearrange the actin microfilaments and promote the breakdown of the actin stress fibers (contractile bundles composed of actin microfilament and NMIIA).

**FIGURE 2:**
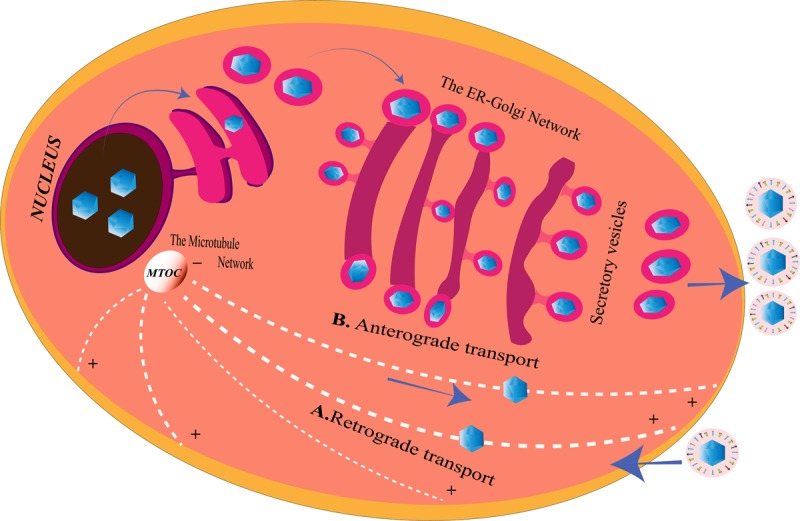
Herpes simplex virus (HSV) transport system inside the cell. **(A)** *Retrograde transport*. After entering the cell, microtubules with the help of the motor proteins transport the virus along the minus end toward the microtubule organizing center (MTOC), from where they enter into the nucleus for genome replication. HSV may also use the endoplasmic reticulum (ER)–Golgi network to travel from the cell membrane to the nucleus for the same since ER shares a continuous membrane connecting the perinuclear space (PNS) and the Golgi apparatus. **(B)** *Anterograde transport*. After replication, the viruses are transported from the nucleus to the MTOC, from the minus end to the plus end of the microtubules to reach the cell membrane from where they are released. The progeny viruses may also travel through the PNS into the ER. The nucleocapsids enclosed in ER membranes are transported on the GA where it is transported along the *trans-*Golgi network into vesicles and released at the cell membrane, a process similar to the release of the secretory proteins of the cell.

Herpes simplex viruse relies on the host transport machinery for its transport to the nucleus. Microtubules, one of the components of the cytoskeleton, play an important role in HSV transport from the plasma membrane to the nucleus (Dohner et al., 2005). This transport is driven by the motor proteins, kinesin and dynein (Lyman and Enquist, 2009). HSV is capable of employing a plus end-tracking protein (+ TIP) complex to begin a retrograde transport toward the nucleus (Figure 2). This complex is made up of end-binding protein 1 (EB1), a cytoplasmic linker protein 170 (CLIP-170), and the dynactin-1 (DCTN1) (Jovasevic et al., 2015). HSV retrogradely transports along the minus end of the microtubules toward the microtubule organizing center (MTOC), located beside the nucleus (Dammermann et al., 2003). The recruitment of the motor proteins, dynein, dynactin, kinesin-1, and kinesin-2, is done by the pUS3, pUL36, pUL37, ICP0, pUL14, pUL16, and pUL21, the capsid proteins exposed to the cytoplasm post-HSV entry. Different proteins on the HSV interact with different motor proteins to facilitate the transport process. VP26 is an outer capsid protein of HSV which interacts with RP3 and Tctex1, the dynein light chains (Douglas et al., 2004). Also, UL34 protein [a component of the HSV nuclear egress complex (NEC)] binds to the dynein intermediate chain (Reynolds et al., 2002). The transport of the virus capsids from the nucleus to the periphery of the cell takes place in an anterograde fashion, along the plus-end of the microtubules (Lee et al., 2006) (Figure 2). The capsid protein pUL37 recruits dystonin (BPAG1), which helps in this trafficking (McElwee et al., 2013; Pasdeloup et al., 2013). In tumor cells arising from oncolytic HSV-1 (oHSV) infection, histone deacetylase 6 (HDAC6) could promote the spread of oHSVs by modulating the trafficking of the oncolytic virus particles (OVs) through acetylation of the microtubule (Nakashima et al., 2015). Other than the microtubule network, HSV is capable of exploiting the endoplasmic reticulum (ER)–Golgi network for its transport within the cell.

## The Endoplasmic Reticulum and the Golgi Apparatus

### Herpes Simplex Virus Strategies for Overcoming Endoplasmic Reticulum Stress

It is also extremely appreciable that HSV is powerful enough to exploit the imperative organelle of the cell for its benefit. The largest cellular organelle, the ER, is a complex network extending throughout the cytoplasm of the cell. It is the site for protein synthesis [rough ER (RER)], modification, and transport of membrane, as well as secretory proteins. As a major organelle for protein folding (essential, as folding provides protein functionality), dysregulation in the ER is capable of changing the entire biology of the cell and drive the cell toward death. ER has a threshold to this folding, and too much protein load forces the ER to cause misfolding of the proteins triggering the unfolded protein response (UPR) pathway. Thus, ER possesses stress-sensing molecules, which regulate the amount of protein taken up for folding. This is because UPR delays the protein folding process, until the protein folding capabilities of the ER would be replenished (Harding et al., 2002). The activation of the UPR pathway may lead to response from either of the two branches. The IRE1/ATF-6 branch directly regulates the ER folding by expressing genes that hold forth the ability of the ER to correctly fold the proteins. The other branch is represented by the kinases which cause a temporary halt in the translation process by phosphorylating and thereby inactivating the translation initiation factor, eIF2α. The two branches of the UPR converge to maintain homeostasis; a counterattack to any stress forced upon the ER (Harding et al., 2002). One of these stresses is virus infection. Viruses require eIF-2 for the production of its own proteins (Liu et al., 2004). However, the phosphorylation of eIF-2 at the α subunit prevents the conversion between guanosine triphosphate (GTP) and guanosine diphosphate (GDP). This further inhibits the recycling process to maintain the production of active eIF-2 for the recruitment of tRNA to the 40S ribosome (Mohr, 2006). Being dependent on the host cell machinery for its life processes, viruses rely on the ER for their protein folding. This creates a stressful condition within the ER where the ER is loaded with a lot of proteins for proper folding, the cellular plus the viral proteins, thus leading to the activation of UPR (Harding et al., 2002). Protein kinase R (PKR) is a component of the UPR that phosphorylates eIF2α. In the early infection stage, HSV-1 shuts off the host gene expression, limiting the load of cellular proteins entering the ER for folding (Harding et al., 2002). Also, products of viral genes Us11 and γ_1_34.5 are able to alleviate the phosphorylation on eIF2α to continue the production of proteins (He et al., 1997; Novoa et al., 2001). It is important to note that Us11 and γ_1_34.5 mutants of HSV-1 can resist acute ER stress, shedding light on the possibility of other mechanisms for blocking the UPR (Mulvey et al., 2006). Thus, the importance of PERK (PKR-like endoplasmic reticulum kinase) in the resistance of UPR was established (Mulvey et al., 2007). HSV-1 uses its gB to accumulate viral polypeptides inside the host cell. The glycoprotein gB of HSV-1 was found to interact with the luminal domain of PERK, the domain that is responsible for recognizing ER stress. A possible mechanism suggested by Mulvey et al. (2007) for the interaction of gB with PERK is similar to the interaction of gB with the MHC-II to disrupt the processing of proteins in the infected cells. The luminal domains in IRE1 and PERK are alike, and both sense the unfolded proteins by the formation of oligomers in these domains (Harding et al., 1999; Bertolotti et al., 2000). These oligomers in PERK create a groove which resembles the peptide binding groove of the major histocompatibility complex (MHC) (Credle et al., 2005). Since gB has an affinity for such a groove in the MHC-II, which it uses to disable the protein processing pathway (Sievers et al., 2002; Neumann et al., 2003), it is possible that gB interacts with the luminal side of PERK in the same way and blocks the ability of PERK to perceive ER stress. PERK remains inactive during HSV-1 infection (Mulvey et al., 2007).

### Herpes Simplex Virus Remodels the Endoplasmic Reticulum–Golgi Structure for Its Survival

Viruses are capable of modifying the membranes of the cellular organelles in the host (Suhy et al., 2000; Tolonen et al., 2001; Egger et al., 2002). There are mainly two reasons for them to remodel the host cellular organelle membrane. Firstly, viruses that replicate in the cytoplasm of the cell require these modified membranes to make compartments. These compartments are called *replication factories* that assist the synchronized gathering of the viral and cellular components for proficient virus replication and assembly. Secondly, the viruses alter the membranes to create a barrier in order to hide from the cellular immune responses (Suhy et al., 2000; Tolonen et al., 2001; Egger et al., 2002). Although not much is known about the membrane remodeling by HSV-1, the Golgi apparatus (GA) and the *trans*-Golgi network (TGN) have been discovered to be dispersed throughout the cytoplasm in the HSV-1-infected cells (Campadelli et al., 1993). ER is not only involved in protein synthesis but also imparts functionality to the protein by folding it in a correct conformation. It is then the ER which provides the virus with the final products of its genes, the proteins that help the virus establish pathogenesis (Romero-Brey and Bartenschlager, 2016). HSV-1 uses its viral protein UL34 to intensely alter the global ER edifice in order to enter the nucleus for replication (Maeda et al., 2017). The entire ER architecture, along with viral factor UL34 and host membrane protein CD98 heavy chain (CD98hc), was found compressed around the nuclear membrane (NM). UL34 is a component of the HSV–egress complex which is involved in the egress mechanism known as the vesicle-mediated nucleocytoplasmic transport (Roller et al., 2000; Reynolds et al., 2001; Mettenleiter et al., 2013). According to this proposed mechanism, HSV acquires a primary envelopment during egress from the inner NM (INM), de-envelops at the outer NM (ONM), which is fusion with the ONM, to expose into the cytoplasm (Johnson and Baines, 2011; Mettenleiter et al., 2013). UL34 along with UL31 helps in the envelopment process and the compression of the ER around the NM. CD98hc is a cell surface glycoprotein that serves as an amino acid transporter by associating with one of the light chains (Verrey et al., 2004). It associates with integrin β1 and β3 to regulate integrin signaling and hence cell adhesion and migration (Fenczik et al., 1997; Feral et al., 2005; Prager et al., 2007). CD98hc, like UL34 in HSV, is involved in the fusion process of other enveloped viruses such as the Newcastle disease virus, parainfluenza virus type-2, and HIV (Ito et al., 1992; Ohgimoto et al., 1995; Okamoto et al., 1997). Despite being a membrane glycoprotein, CD98hc retention at the plasma membrane (PM) is inhibited by HSV components, causing the accumulation of CD98hc in the ER. UL34 induces the compression of ER around the NM, bringing the CD98hc along with it (Maeda et al., 2017). It can be inferred that the remodeling of the ER by the virus is done to build up an environment of fusion molecules around the NM for efficient egress of HSV. Since the ER–Golgi network is continuous, the GA is also transformed post-HSV infection.

The GA works hand in hand with the ER to modify the proteins to be secreted outside the cells (Figure 2). Therefore, the membrane of GA is an extension of the ER membrane for efficient transport of proteins from the GA to the periphery of the cells (Bauerfeind and Huttner, 1993). As mentioned above, HSV-1, apart from remodeling the ER for its own needs, also disturbs the Golgi integrity (Campadelli et al., 1993). Martin et al. (2017) observed that two players in GA integrity, Src tyrosine kinase and dynamin 2 (Dyn2) GTPase, mediate the disturbance of GA structure post-HSV-1 infection in primary neuronal cells. HSV-1-infected primary neuronal cells depicted activated Src kinase and subsequent phosphorylation of its substrate dynactin 2 to reveal perturbation in the GA structural integrity. This distortion in the organelle structure could be an evidence for the HSV neuropathogenesis through the destruction of the secretory system. Src (pronounced as “sarc” for the short form of sarcoma) protein kinases are non-receptor kinases involved in oncogenesis. They play major roles in cell growth, division, migration, and survival pathways (Roskoski, 2015). Src tyrosine kinases are themselves activated by phosphorylation at Y424 residue and phosphorylates its substrate Dyn2 at the residues Y231 and Y597 (Weller et al., 2010). Phosphorylation of Dyn2 activates its GTPase activity (Cao et al., 2010). The GTPase activity of Dyn2 controls the fragmentation of GA and the TGN during the secretory processes (Ishida et al., 2011). Continued activation of Src kinase and Dyn 2 have been known to disturb the integrity of GA in other cell types as well (Weller et al., 2010). Martin et al. (2017) demonstrated that the viral tegument protein VP11/12 of HSV-1 is not crucial but a partial contributor to the activation of Dyn2 through Src kinases, leading to degradation of the GA during HSV-1 multiplication in the primary neurons.

There are two possible propositions to the mechanism of Src activation in the HSV-1-infected neurons. One possibility is activation through direct HSV interaction with the cellular receptors. As known with other herpesviruses, gH/gL gps initiate the entry process by interacting with αvβ8 integrin and require activated Dyn2 for the same (Gianni et al., 2010). Dyn2 physically interacts with focal adhesion kinase (FAK) to be recruited to the focal adhesion sites. Here, Dyn2 is activated by Src and promotes the induction of endocytosis of the integrins, thus, easing the invasion of the cells (Wang et al., 2011). Another possible mechanism for Src activation is the interaction of the viral proteins with Src, after the entry of HSV. The SH2 domain of the Src is bound by the VP11/12 tyrosine motifs to stimulate the phosphoinositide 3-kinase (PI3K)/AKT pathway in the T cells (Strunk et al., 2016). Therefore, VP11/12 may activate Src kinases post-virus entry in neuronal cells as well, although Src activation through other mechanisms is possible.

### The Endoplasmic Reticulum–Golgi Network as the Carriage for the Transport of Herpes Simplex Virus

Different courses of nuclear egress have been proposed through the years. According to one of those proposed routes, fusion at the ONM is quite a popular one. Fusion at the ONM is supported by the evidences of primary envelopment at the INM, succeeded by de-envelopment or fusion at the ONM. This fusion or the de-envelopment process, where the primary envelop fuses with the ONM, requires gH/gL of the HSV. However, enveloped virions were observed in the cytoplasm as well as the extracellular space in gH/gL deleted mutants, suggesting that there are other departure routes of the HSV from the cell. Wild et al. (2018) remarked that HSV exploits the ER–Golgi transport system of the cell for egress from the NM to the PM and out of the cell. In natural circumstances, the freight is transported from the ER to the GA *via* vesicles budding out of the ER exit positions (Bonifacino and Glick, 2004) or through an ER–Golgi intermediate compartment (EGIC) (Hauri and Schweizer, 1992; Klumperman, 2000; Saraste et al., 2009). In the cisternae of the GA, the freight is parceled in granules to be released outside the cell (Palade, 1975) (Figure 2). The packaging process results in a loss of the GA membrane, but the GA has multiple ways of replenishing it (Orci et al., 1981). After HSV replication and capsid assembly in the host nucleus, the capsids are sent to the GA for the acquisition of the tegument and envelope. The enveloped virion is then covered into a transport vacuole. This enclosing procedure is known as *wrapping* (Roizman et al., 2014). When HSV-1-infected cells were observed between 8 and 16 h post-infection (hpi), the membranes of the GA, ER, and ONM were connected to establish a continuum between the perinuclear space (PNS) and the Golgi cisternae (Figure 2). The number of virions in the ER was increased to almost four times by the end of 24 hpi after which the GA was degraded (Wild et al., 2015). Therefore, GA integrity is important for the transport of virions out of the ER. The path of HSV transport is similar to the transport of secretory proteins out of the cell, which is through vesicle formation or EGIC (Klumperman, 2000). Also, the intraluminally transported virions are coated with a dense proteinaceous layer. The layer may be used as a protection against the fusion of the viral membrane with the transport organelle membranes, thus strengthening the possibility of an alternative route for HSV egress. The translation of HSV proteins is an important step before the packaging of viral particles. Ribosomes, being a part of the RER and site of viral translation, are also manipulated by HSV for its pathogenesis.

## Ribosomes

### The Role of the Ribosomal and Viral Proteins in Herpes Simplex Virus Translation

Ribosome is the cellular factory for the production of proteins; a process known as “translation” in the central dogma. It contains ribosomal RNAs (rRNAs) that catalyze the peptide bond formation between the amino acids and ribosomal proteins (RPs) to regulate the translation process. The formation of ribosomes in eukaryotes (80S) takes place in the nucleolus. The eukaryotic 80S ribosome is composed of a small subunit (40S) and a large subunit (60S). The 40S subunit, known for decoding the mRNA for the incorporation of the appropriate amino acid, is made up of the 18S rRNA and 33 RPs. The 5S, 5.8S, and the 28S rRNA along with around 47 RPs assemble into the 60S subunit that catalyzes the peptide bond formation. Therefore, the eukaryotic ribosome is composed of four rRNAs and about 80 RPs (Wilson and Doudna Cate, 2012). RPs, such large in numbers, function as chaperones to stabilize and facilitate the correct folding of the rRNAs for ribosomal assembly (Fromont-Racine et al., 2003). The RPs are also important in the regulation of cell proliferation, cell cycle, apoptosis, tumorigenesis, development, and genome integrity (Chen and Ioannou, 1999; Bhavsar et al., 2010; De Las Heras-Rubio et al., 2014; Xu et al., 2016). During some viral infections, such as those with herpesviruses, the translation of cellular mRNA transcripts may be halted or remarkably decreased, whereas that of RP mRNAs are increased and persisted late to sustain the propagation of virus (Greco et al., 1997; Simonin et al., 1997). L22, an RP, has been known to interact with infected cell protein 4 (ICP4). The ICP4 protein of HSV-1 is an immediate early protein and a transcription regulator of many of the early and the late HSV-1 genes required for the synthesis of viral DNA and increasing pathogenesis in the host (Leopardi and Roizman, 1996; Li, 2019).

**FIGURE 3:**
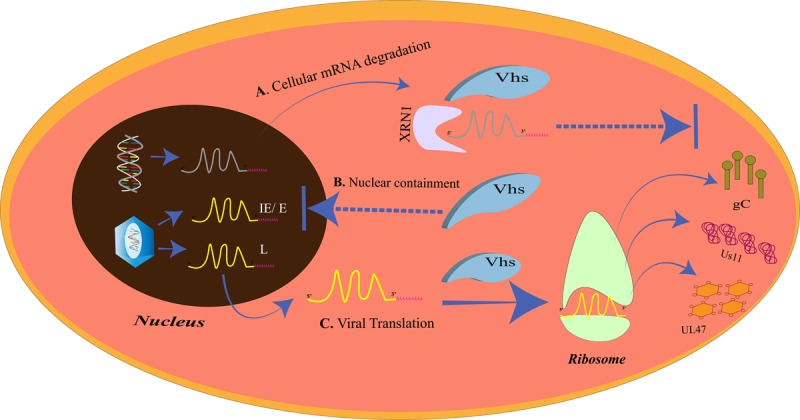
The virion host shutoff (vhs)-mediated translational regulation. **(A)** *Cellular mRNA degradation*. The vhs protein triggers the degradation of the cellular mRNAs by XRN1. **(B)** *Nuclear containment*. Vhs also prevents the mRNA overloading at the ribosomes at the time of late gene expression by restricting the IE/E viral mRNAs in the nucleus. **(C)** *Viral translation*. Cellular mRNA degradation and nuclear containment of other IE/E mRNAs allows only the late viral mRNAs to exit the nucleus and be translated at the ribosomes. The late gene proteins, gC, Us11, and UL47, inhibit the host immune complement system, prevent autophagy, and promote nuclear egress, respectively.

Initiation of translation of the mRNA transcripts to proteins requires the recruitment of the cap binding complex eukaryotic initiation factor 4F (eIF4F), which is composed of eIF4E, eIF4G, eIF4A, eIF4B, and eIF4H, to the 5′m7G-cap of the mRNA. eIF4G in the bound state staffs the assembly of the 43S preinitiation complex. The pre-initiation complex which mainly constitutes the 40S ribosomal subunit moves over the 5′ untranslated region (UTR) to trace the start codon. The joining of the 60S ribosomal subunit to the pre-initiation complex creates a functionally active 80S ribosome, ready for translation (Jackson et al., 2010). Like other biological processes, HSV is dependent on the translation machinery of the host for the translation of around 70 encoded proteins. Therefore, it would always try and make the translation process more efficient and unhindered through a number of strategies (Smith et al., 2008; Walsh and Mohr, 2011). One such strategy is the enhancement of the translation initiation by increased assembly of the cap binding complex. The ICP6, ICP27, and the HSV Ser/Thr kinase US3 are the three proteins responsible for the improved assembly of the cap binding complex (Walsh and Mohr, 2004; Hargett et al., 2005; Walsh and Mohr, 2006; Chuluunbaatar et al., 2010). For efficient translation of its own proteins, HSV may paralyze the host cellular gene expression such that more of its mRNA transcripts gain access to the translation machinery. HSV encodes the virion host shutoff (vhs) protein which acts as an endonuclease and is an mRNA-specific destructor. Vhs is essential for the translation of late mRNAs of HSV-1 (Dauber et al., 2014) (Figure 3). It is encoded by the UL41 gene and is a tegument protein. Vhs destabilizes the cellular mRNAs by associating with helicase and helicase cofactors (Doepker et al., 2004; Feng et al., 2005; Page and Read, 2010; Read, 2013; Shiflett and Read, 2013). The mRNA degradation is assisted by the cellular RNase, XrnI (Gaglia et al., 2012). Vhs-mediated mRNA destruction achieves two goals for the virus at the same time. Firstly, it reduces the competition among the mRNA transcripts to be translated by the host translation machinery and, secondly, cripples the antiviral immune response of the host (Paladino and Mossman, 2009). Although it might seem odd, vhs destabilizes viral mRNAs as well (Read, 2013). Such destabilizations are vital for the shifts between the immediate early, early, leaky late, and true-late HSV gene expression, so that the competition for the accessibility toward the translation apparatus is further reduced. Nevertheless, the translation of the true-late mRNA transcripts, US11, UL47, and gC, was impaired in vhs-deficient HSV-1-infected Hela cells. The levels of US11 could be restored if the US11 late-mRNA transcripts are present before the ribosomes and the factors required for translation become limiting. These results suggest that in the absence of vhs, the translation machinery is overwhelmed with mRNAs of both the host and HSV and will not translate the mRNAs that have entered later that is the mRNAs of late-viral genes (Dauber et al., 2014). Our cell, however, has its own mechanism of “self-clearance” of these viral proteins, which is housed by the lysosomes of the cell.

## Lysosomes

### Importance of “Self-Clearance” in Cytoprotection

**FIGURE 4:**
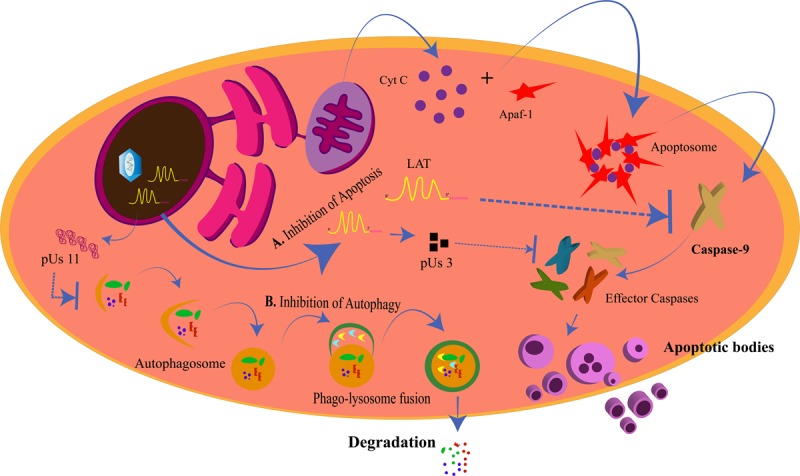
Inhibition of apoptosis and autophagy by herpes simplex virus (HSV). **(A)** *Inhibition of Apoptosis*. The intrinsic apoptotic pathway for the programmed death of a cell is generated from the mitochondria when cytochrome C is released and forms the apoptosome along with Apaf-1 and other proteins. This allows for the activation of caspase-9 and the other effector caspases to generate the apoptotic bodies. LAT and pUs 3 block the activation of caspase-9 and the effector caspases, respectively, to prevent the cell from dying for its own survival. **(B)** *Inhibition of Autophagy*. pUs 11 inhibits the process of autophagy through which the enzymes from the lysosomes degrade the viral proteins, hence inhibiting viral clearance from the cell.

Lysosomes are intracellular membrane-bound compartments that contain digestive enzymes for the degradation of unwanted contents in the cytoplasm such as toxic, defective, or excess proteins, bacteria, and viruses. This degradation process is called *autophagy* and is triggered in response to a pathogen attack, starvation, stress, and hypoxia, with more than 35 proteins (ATG) directing the process. This way, autophagy by lysosomes is a mechanism of cleansing of the cell to increase its longevity. The steps in autophagy can be chronologically stated as: (1) phagophore initiation; (2) elongation of the membrane; (3) formation of the autophagosome; (4) fusion of the autophagosome with the hydrolytic enzymes in the lysosome (Mizushima et al., 2011). The targets of autophagy are selected depending upon the selective receptors expressed on the phagophore. These receptors have ubiquitin binding domain (UBD) to interact with the ubiquitin tags on the targets and LC3-interacting region (LIR) motif that interacts with the LC3 proteins (Stolz et al., 2014). Tank-binding kinase 1 (TBK1) is a regulator of autophagy and is instrumental in the destruction of pathogens by the lysosome (Wild et al., 2011; Pilli et al., 2012; Sparrer et al., 2017). In case of a viral attack, the process of autophagy could be modulated by the virus for its own survival or can be simply evaded by the expression of specific proteins by the virus. The virus needs to avoid the autophagic process when autophagy is protective to the cell in various ways. The cytoprotective strategies of autophagy are: targeting pathogens for destruction, promoting and/or regulating inflammation, promoting antigen presentation, and spreading protection *via* autophagy to the neighboring cells (Levine, 2005; Levine et al., 2011; Jackson, 2015; Paul and Münz, 2016). HSV-1 is such a virus that inhibits the protective effects of autophagy. Primary neuronal cells in mice choose autophagy over interferon-mediated antiviral effects to eliminate the virus (Yordy et al., 2012). HSV-1 inhibits autophagy by the action of US11 and ICP34.5 proteins (Figure 4). US11 dephosphorylates PKR and inhibits the phosphorylation of eIF2α; phosphorylated eIF2α being the inducer of autophagy (Lussignol et al., 2013). ICP34.5 interacts with beclin-1 and inhibits autophagy (Orvedahl et al., 2007).

Autophagy can allow the cell to survive for longer periods of time by obstructing pathogenesis. Mouse mutant cells having high basal autophagy levels restricted HSV-1 replication (Le Sage and Banfield, 2012). Moreover, evidence shows that drugs that induce autophagy are capable of reducing viral loads. MG132 is one such inducer that can decrease the HSV-1 titers in human corneal epithelial cells (Yakoub and Shukla, 2015). Similarly, rapamycin could restrict the pathogenesis of HSV-1 in human fibroblast cells and promote the survival of the cells (Ahmad et al., 2019). The protective autophagic effect in the cells with HSV-1 infection has been attributed to TBK-1. TBK-1 is a cellular kinase that phosphorylates and activates the selective receptors on the phagophore for the selective targeting of the cargo to be carried to the lysosome for destruction by the hydrolytic enzymes (Ahmad et al., 2019). Antiviral responses such as those from interferon (IFN)γ are significantly reduced when the MHC-II antigen presentation is reduced. Autophagy can allow HSV-1 antigens to be cross-presented to MHC-I. Studies have shown that the HSV-1 gB could be cross-presented on MHC-I of the BMA3.1A7 macrophage in an autophagy-dependent fashion (English et al., 2009; Radtke et al., 2013). Also, cells infected with HSV-1 could be induced to show autophagic effects *via* TBK-1-mediated paracrine signaling. The paracrine-mediated autophagy occurs early in the HSV-1 infection and protects the infected cell from dying (Ahmad et al., 2019). Another mechanism of viral clearance is the programmed death of the cell to destroy itself and the pathogen within it to restrict the viral spread.

## Mitochondria

### Herpes Simplex Virus Manipulates the “Power House” of the Cell

Another cellular organelle that may share a common membrane with the ER is mitochondria. Mitochondria, known as “*the power house of the cell*” as they are the main compartments for the production of ATPs, are dispersed throughout the cytoplasm of the cell. Mitochondrion has its own genome, replication equipment, and transcription/translation machinery but is also dependent on the nuclear genes, without the expression of which it cannot function actively. Also, if the mitochondria fail, the cell cannot survive. This is because apart from energy synthesis, mitochondria are involved in a number of cell processes such as apoptosis and regulation of calcium levels, which affect the survival of the cell. Hence, the mitochondrial changes induced by a viral attack are critical for the cell (Murata et al., 2000).

### Maintenance and Exploitation of Mitochondria Until Mid-Herpes Simplex Virus Infection, Followed by Its Degradation at the Later Stages

Herpes simplex viruse infection of the cell has been known to degrade the mitochondrial DNA (mtDNA) rapidly and completely by an HSV nuclease, UL12 gene product (Saffran et al., 2007). This is in compliance with the early findings where the production of mitochondrial proteins decreased by 60% in HSV-infected cells when compared to the uninfected cells (Lund and Ziola, 1986; Latchman, 1988). In another study, the mitochondrial damage has been associated with encephalitis caused by HSV infection. Neuronal cells have confirmed the severe destruction of mitochondrial mRNA transcripts and mtDNA through the pUL12.5 or US3 viral proteins of HSV (Wnêk et al., 2016). The cytochrome C oxidase (CO), the last enzyme in the electron transport chain, was markedly decreased in astrocytes at 24 hpi (Wnêk et al., 2016). Murata et al. (2000) found that the mitochondria gathered around the nucleus along with the tegument proteins UL41 and UL46, post-HSV infection. HSP60, a protein responsive to stress, was found elevated in such conditions. ATP and lactate levels in the cells were maintained up to 6 hpi but decreased later on, which indicates that mitochondria are responsive to HSV infection. They migrate with the tegument proteins to the PNS, forming a ring-like structure around one side of the nucleus, functioning optimally until the mid-infection stage after which the mitochondrial integrity decreases. The presence of mitochondria in its condensed state around the nucleus, where the mitochondria are highly active for respiration, is essential when the HSV morphogenesis is under process, so that the morphogenesis process is provided with an ample supply of energy *via* ATP (Murata et al., 2000).

### Modulation of Apoptosis by Herpes Simplex Virus

Mitochondria are involved in the process of apoptosis. Programmed cell death or apoptosis is critical for the cell as it decides the fate of the cell. The process is well-defined and causes the destruction of cells to promote development or inhibit the spread of an infection or growth of a cancerous tissue. Cells undergoing apoptosis are distinguished by shrinkage, formation of apoptotic bodies, and nuclear fragmentation (Wyllie, 1997). The two pathways of apoptosis, the intrinsic and the extrinsic pathways, converge at the activation of cysteine-specific aspartate protease (caspase) enzymatic pathway causing proteolysis and ultimately death of the cell (Galluzzi et al., 2012). The *intrinsic pathway* is so called due to the involvement of the cell’s own component, the mitochondria. The mitochondrial pathway is initiated with the triggering of the mitochondrial outer membrane permeabilization (MOMP). This allows the exit of the cytochrome C from the inner mitochondrial membrane to the cytoplasm through the pores in the mitochondrial membrane. The apoptotic pathway is regulated by the members of the Bcl-2 protein family. Cytochrome C binds to Apaf-1 to initiate the apoptosome assembly. The apoptosome recruits procaspase-9. Procaspase-9 is cleaved to generate the active caspase-9. Thus, the caspase cascade is triggered with the subsequent activation of the other caspases for complete destruction of the cell (Yuan and Akey, 2013; Yuan et al., 2013; Jiang, 2014). PERK, which is an ER stress responsive protein, is also a contributor to the intrinsic apoptotic pathway (Verma and Datta, 2012). Apoptosis of virus-infected cells restricts replication and transmission of the viruses. Hence, HSV tries to inhibit apoptosis in the infected cells (Figure 4). The anti-apoptotic proteins of HSV are US3, gJ, and latency-associated transcript (LAT). The LAT is transcribed and spliced during HSV latency and is an inhibitor of apoptosis in the infected cells (Wagner et al., 1988; Jones, 2013). It inhibits caspase 8/9-mediated apoptosis (Henderson et al., 2002) by the maintenance of phosphorylated levels of AKT, which in turn phosphorylates to inactivate the pro-apoptotic proteins (Bad, Bax, caspase 9) (Liu and Cohen, 2015). The LAT gene is 8.3 kb of which the initial 1.5 kb transcribes into two small RNAs of 62 and 36 nucleotides, those that are responsible for the anti-apoptotic effects of LAT (Shen et al., 2009). On one hand, where LAT is capable of preventing apoptosis even when the other HSV-1 genes are absent (Carpenter et al., 2007), ICP22 is not a very strong anti-apoptotic protein. ICP22 is a regulator of the expression of the anti-apoptotic genes of HSV-1 and does not directly interfere with the apoptotic signaling (Aubert et al., 2008). ICP22 inhibits the pro-apoptotic functions of p53 by alleviating the inhibitions on Bax (Pietsch et al., 2008). The C- terminus of ICP27 is also an indirect inducer of anti-apoptotic effects, increasing the anti-apoptotic gene expressions (Fontaine-Rodriguez and Knipe, 2008) and also promoting the activation of nuclear factor (NF)κB, encouraging cell survival (Hargett et al., 2006). US3 protein kinase is another direct inhibitor of procaspase 3, impeding the mitochondria-mediated apoptotic pathway (Benetti and Roizman, 2007). The viral protein US3 is also involved in the egress of HSV from the nucleus. The nucleus being the “brain of the cell” is one of the major target organelles for the virus.

## Nucleus

### Temporal Retention of the Herpes Simplex Virus Genes to Avoid Load on the Host Machinery

Nucleus is the compartment for cellular and viral genome replication as well as the transcription of mRNA from the genetic information. Since the genetic code for all life processes is contained in the nucleus, the nucleus is also known as the “information center” of the cell. The mRNA transcripts leave the nucleus when they are needed to be translated into proteins. Vhs, the HSV host shutoff protein responsible for cellular mRNA degradation, is also responsible for the retention of viral mRNAs in the nucleus (Elliott et al., 2018). Vhs causes the retention of the IE and the E mRNA transcripts in the nucleus but allows the late transcripts to translocate to the cytoplasm. This occurs at the beginning of the late gene transcription. As a regulator of vhs, VP22 is able to release the vhs-induced nuclear retention on the late transcripts, to permit their translocation to the cytoplasm, so that the late proteins could be translated from them. HSV-1 checks the load of transcripts entering the translation machinery for the efficient progression of infection by not only restricting the cellular mRNAs but also its own mRNAs (Pheasant et al., 2018).

### The Nuclear Envelope Disruption Model for Herpes Simplex Virus Egress

**FIGURE 5:**
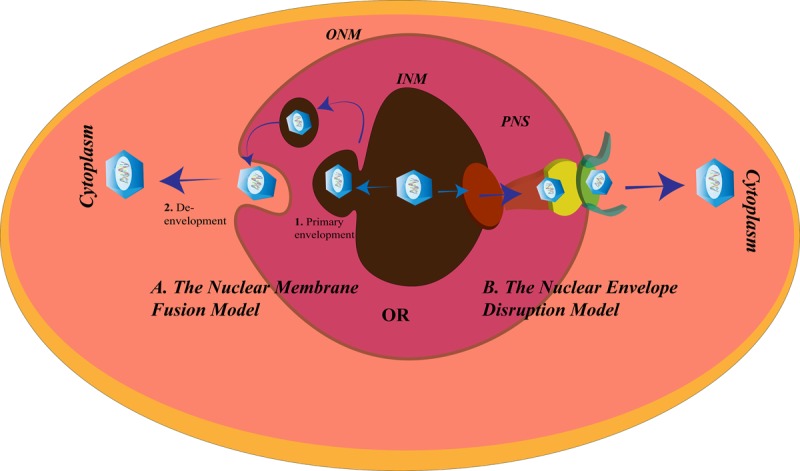
Herpes simplex virus (HSV) nuclear egress. There are two proposed mechanisms for the exit of HSV from the nucleus of the cells. **(A)** *The nuclear membrane fusion model* in which the HSV nucleocapsid acquires the inner nuclear membrane (INM) envelope (1. Primary envelopment) and is released into the perinuclear space (PNS), followed by fusion of this acquired envelope with the outer NM (ONM) to allow the exit of the nucleocapsid from the nucleus and enter the cytoplasm (2. De- envelopment), and **(B)** *the nuclear envelope disruption model* in which the lamins underlying the INM are modified and the nuclear pore disrupted to make way for the exit of the HSV nucleocapsid into the cytoplasm.

The nuclear envelope (NE) serves as a barrier for the viruses; the second gateway for the HSV to pass through (Figure 5). The NE of the nucleus is divided into three regions: the ONM, the PNS, and the INM. The gap between the ONM and the INM is the PNS with a diameter of 20–50 nm and is continuous with the ER. The PNS is spanned by the nuclear pore complexes (NPCs) connecting the nucleus to the cytoplasm (Stewart et al., 2007). Assembly of the HSV-1 capsids takes place in the nucleus. After assembly, it is either transported to the PNS and buds off at the ONM or impairs the nuclear pore (protein channels) to create distortions in the NE to exit the nucleus (Leuzinger et al., 2005; Wild et al., 2005). Beneath the INM, toward the nucleoplasm, is the nuclear lamina. The function of the nuclear lamina is to provide structural sustenance to the NE, hence, it is made up of an assembly of lamin A/C/B along with the membrane proteins. The HSV nucleocapsids, being larger in size (120–130 nm in diameter) than the lamin network (with spacing of about 15 nm) or nuclear pores (with a diameter of about 38 nm), need a different mechanism to modify the lamina and the NE to exit from the nucleus (Alber et al., 2007; Goldberg et al., 2008; Lee and Chen, 2010). One such mechanism is the interaction of the nuclear HSV egress complex (pUL31 and pUL34) with lamin to disrupt the further associations between the lamins (Reynolds et al., 2004). Another mechanism is the hyperphosphorylation of emerin, a membrane protein. PKC-δ and US3 hyperphosphorylate the emerin to disturb its interaction with the lamins (Leach et al., 2007; Morris et al., 2007). Although PKC-α, but not PKC-ζ, is also recruited to the NE after HSV-1 infection, PKC-α is not uniquely required for replication of HSV-1 (Leach and Roller, 2010). The US3 kinase is also involved in the alterations of the nuclear pore for virus egress. Wild et al. (2019) demonstrated the role of US3 in nuclear pore impairment post-HSV infection. Confocal super resolution microscopy and cryo-field emission showed significant loss of nuclear pores in HSV-infected cells, and this extreme decrement was not observed in US3-deleted variants of HSV. Moreover, the maximum numbers of capsids were retained in the nucleus in US3-deleted mutants and very minimum were observed in the cytosol, whereas the opposite scenario was observed in the wild-type HSV-1 infection. Thus, it was concluded that US3 is vital in damaging the nuclear pore of the host nucleus for the egress of HSV (Wild et al., 2019).

### The Nuclear Membrane Fusion Model for Herpes Simplex Virus Egress

According to an alternative route for nuclear egress, HSV leaves the nucleus *via* the membrane fusion mechanism (Figure 5). There are two steps according to the fusion model:

(1) *Primary envelopment*, where the newly formed nucleocapsids bud from the INM. During this process, they acquire the membrane components of the INM and become enveloped. After budding, the enveloped HSV particles are present in the PNS. The NEC comprising the HSV protein products of the gene UL31 and UL34 assists the primary envelopment. Without the NEC, the nucleocapsids are retained in the nucleus (Reynolds et al., 2001). Other proteins encoded by the HSV may be recruited by the NEC to help them in the primary envelopment. HSV-1 encodes UL41 and ICP22 that were found to be co-localized with the NEC at the INM to assist the NEC by interacting with the proteins involved in HSV-1 nuclear egress (Liu et al., 2014; Maruzuru et al., 2014). UL16 and UL21 of HSV-2 are known to contribute to its primary envelopment (Le Sage et al., 2013; Gao et al., 2017).

(2) *De-envelopment*, where the HSV envelope fuses with the ONM to release the nucleocapsids into the cytoplasm. Like the fusion at the cell membrane, the fusion between the primary virions and ONM, involves glycoproteins. In the absence of gB and gH, HSV-1 fails to exit from the NE (Farnsworth et al., 2007). pUS3 mediated phosphorylation of pUL31, and gB is essential for the fusion process (Mou et al., 2009; Wisner et al., 2009). Also, recruitment of CD98hc, p32, and incorporation of β1 integrin to the membrane of the nucleus after HSV-1 infection is supportive of the fusion model (Hirohata et al., 2015; Liu et al., 2015). Failure to recruit any of these proteins causes accumulation of the virions in the PNS or vesicles derived from INM.

## Conclusion

The entry of the virus into the host cells can be considered as the most important step in the HSV-1 infection cycle. This is because HSV-1 needs to enter the cell in order to begin its replicative cycle. Hence, small molecules, peptides, or nanoparticles that can block the HSV-1 entry into the cells might prove to be strong antiviral candidates. One such small molecule is epigallocatechin gallate that competitively inhibits the binding of HS to HSV-1 (Colpitts and Schang, 2014). Certain small, cationic peptides have been recognized as HSV-1 attachment inhibitors and block HSV-1 entry (Tiwari et al., 2011; Jose et al., 2013; Jaishankar et al., 2015). BX795, a TBK-1 inhibitor, lowers ocular HSV-1 infection by inhibiting the Akt pathway, ultimately blocking the HSV-1 protein synthesis. This kinase inhibitor is being considered at par with trifluorothymidine (TFT), which is the currently prescribed therapeutic for ocular herpes (Jaishankar et al., 2018). Yadavalli et al. (2019) have found activated carbon particles to be efficient acyclovir (ACV)-drug delivery systems that trapped the virions within themselves. Zinc oxide micro-nanoparticles and nanowires can inhibit HSV-1 entry as well (Antoine et al., 2012; Trigilio et al., 2012). Such nanowires can block HSV-1 cell-to-cell spread. With respect to the cytoskeleton remodeling by HSV-1, there have been inhibitors of the microtubule, such as nocodazole (Naranatt et al., 2005), but they have not proven to be much successful antivirals. It may also be brought to our attention that the complete trafficking process which leads to cancer has not yet been elucidated. High-resolution imaging such as atomic force microscopy (AFM) can reveal the ultrastructure of the tumors caused by HSV infection (Deng et al., 2018). This might shed some more light on the formation of tumors by the manipulation of the cytoskeleton by viruses. Autophagy in HSV-infected cells may impart protection to certain cell types by restricting the HSV load in these cells and allowing the cells to survive. Therefore, HSV tries to inhibit autophagy. In neuronal cells, where HSV prefers to hide itself from the immune cell attacks from the host, autophagy-enhancing agents can be selected as the appropriate therapeutics for decreasing the HSV infection (Yakoub and Shukla, 2015; Ahmad et al., 2019). Similarly, HSV does not allow the cell to die through apoptosis, or else its only life support system would be destroyed. Hence, HSV puts in a lot of effort to inhibit apoptosis of the target cells. Inhibiting apoptosis is a very effective mechanism while establishing a latent infection (Jones, 2013). Therefore, a clarification of these anti-apoptotic effects of HSV can lead to the development of drugs that can promote cell death of the infected cells at the early stage of the infection, exposing HSV to the host immune attacks. Also, the cellular morphology is affected in post-HSV infection. Membrane-bound organelles such as ER and mitochondria are compressed around the nucleus for the proper recruitment and accessibility to the factors required for HSV egress. Thus, this review gives an idea about HSV–host cell interaction and how the knowledge of these interactions would help bridge the gaps in HSV research. It is evident that HSV, being a virus that has co-evolved with humans, is capable of exploiting the cellular organelles for increasing its pathogenesis. Whether it be the rearrangement of the organelle’s membrane (cell membrane, ER–Golgi, mitochondria, nucleus) or inhibiting the counterattacks from the organelles (autophagy, apoptosis, ER stress), HSV successfully overpowers the host cell. Further investigations in unraveling these mechanisms deeper at the molecular level would open up new avenues in the discovery of drugs or vaccines against HSV that would be more effective than the present ones.

## Author Contributions

AB and AM conceptualized the review. AB wrote the article and made the figures. SK was involved in revision. AM edited and supervised the manuscript.

## Conflict of Interest

The authors declare that the research was conducted in the absence of any commercial or financial relationships that could be construed as a potential conflict of interest.
